# The modulation of event-related alpha rhythm during the time course of anticipation

**DOI:** 10.1038/s41598-019-54763-1

**Published:** 2019-12-03

**Authors:** Marie Simonet, Hadj Boumediene Meziane, Oliver Richard Runswick, Jamie Stephen North, Andrew Mark Williams, Jérôme Barral, André Roca

**Affiliations:** 1 Institute of Sport Sciences, University of Lausanne, Lausanne, Switzerland; 2 Institute of Psychology, Faculty of Social and Political Sciences, University of Lausanne, Lausanne, Switzerland; 3 Institute of Sport, University of Chichester, Chichester, UK; 4 Expert Performance and Skill Acquisition Research Group, Faculty of Sport, Health, and Applied Science, St Mary’s University, Twickenham, London UK; 5 Department of Health, Kinesiology, and Recreation, University of Utah, Salt Lake City, UT USA

**Keywords:** Neuroscience, Cognitive neuroscience

## Abstract

Anticipation is the ability to accurately predict future actions or events ahead of the act itself. When attempting to anticipate, researchers have identified that at least two broad sources of information are used: contextual information relating to the situation in question; and biological motion from postural cues. However, the neural correlates associated with the processing of these different sources of information across groups varying in expertise has yet to be examined empirically. We compared anticipation performance and electrophysiological activity in groups of expert (n = 12) and novice (n = 15) performers using a video-based task. Participants made anticipation judgements after being presented information under three conditions: contextual information only; kinematic information only; and both sources of information combined. The experts responded more accurately across all three conditions. Stronger alpha event-related desynchronization over occipital and frontocentral sites occurred in experts compared to the novices when anticipating. The experts relied on stronger preparatory attentional mechanisms when they processed contextual information. When kinematic information was available, the domain specific motor representations built up over many years of practice likely underpinned expertise. Our findings have implications for those interested in identifying and subsequently, enhancing the neural mechanisms involved in anticipation.

## Introduction

The ability to accurately anticipate future events is crucial in dynamic environments where split-second decisions are needed. These dynamic environments comprise domains as diverse as emergency medicine, sport, aviation, law enforcement, and driving^1–4^. In such situations, performers not only operate under strict time constraints, but must frequently make anticipation judgments under psychological and physiological stress that repeatedly challenges the limits of their capabilities. The amount of practice accumulated over years of engagement has consistently been shown to be associated with the development of key numerous perceptual-cognitive skills that underpin anticipation^5^. While successful anticipation is dependent on the ability to pick up accurate information from the display within the time available to respond, the importance of contextual information, often not directly present in the display, has recently been highlighted^6–10^. Yet, to date, scientists have largely overlooked the neural processes that underpin the ability to anticipate^11^. In the current study, we investigated the electrical neuroimaging dynamics that underpin the use of contextual and kinematic information during anticipation in a dynamic and time-constrained task.

The ability to pick-up visual sensory information from the environment (usually via biological motion^12–15^ or perception of patterns between features^16,17^) has been reported to underpin anticipation. Film-based temporal and spatial occlusion methods have been consistently employed to identify the time and location of information pick-up across many domains, including music^15^, driving^1^, team sports^18–20^ and interceptive tasks^21^. An additional source of information that has been reported to facilitate anticipation is the ability to understand the broader context of the situation. McRobert and colleagues^3^ demonstrated that when contextual information such as the patient’s medical history or the results of previous medical test was available, skilled physicians were better able to predict future medical events. It was proposed that through their extended engagement with these environments and tasks, skilled physicians developed domain-specific knowledge skills, which allowed them to integrate such contextual information with previously experienced situations to inform accurate diagnoses. Similarly, skilled athletes have been shown to utilise contextual information that is available prior to the emergence of key postural cues to inform accurate judgments of what might happen next^22^. This contextual information can include, for example, an opponent’s action preferences, the field positions of teammates and opponents, and the score in the game^8,22–25^.

Sport, given its dynamic nature, the ongoing interaction between different performers, and the time constraints under which decisions must be made, provides an ideal vehicle for scientists to investigate expert anticipation. In the current study, we used the sport of cricket as a vehicle to investigate anticipation. Cricket requires batters to pick-up relevant information under severe time constraints in order to predict the trajectory of a ball travelling at high velocity before executing an accurate interceptive action. Scientists have employed gaze recording and think aloud protocols to show that skilled batters are able to use kinematic information from the opponent’s postural orientation ahead of the ball leaving the bowler’s hand^22^. Additionally, presenting extra contextual information such as the score in the game, the field settings or past action outcomes prior to the postural cues becoming available, further improves anticipation accuracy^10,22,25,26^. While these researchers have used video-based tasks and manipulated access to contextual information and/or biological motion cues, in conjunction with collecting process-tracing measures such as gaze data and verbal reports, no researchers have recorded neurophysiological measures simultaneously to investigate the neural correlates of skilled anticipation.

Several scientists have reported that anticipation in sport^27–32^ and aviation^33,34^ is driven by differences in neurophysiological function across different skill groupings. Moreover, in order to examine how different sources of information are integrated during anticipation, Gredin and colleagues^35^ explored the time-frequency modulations when processing contextual priors during a video-based anticipation task involving soccer simulations. Their findings, which showed that frontal theta and parietal alpha ratio was higher when contextual priors were displayed than in the control condition, provided neurophysiological evidence of an increased demand on cognitive processes when analysing contextual information during anticipation. Also, in an effort to explore how visuospatial information is integrated, a recent study showed that superior anticipation in tennis was associated with stronger event-related desynchronization in the mu and beta frequency bands over left sensorimotor areas in skilled compared to novice players when watching tennis footage^11^. The authors argued that for skilled players brain regions involved in supporting action execution show greater activity when engaged in action observation. Consistently, mu suppression over central electrodes has been reported in expert pilots when presented with images of typical landing scenarios^34^. This pattern of neural activity was associated with sensorimotor mechanisms involved in well-known situations where embodied motor representations are activated when observing these previously experienced situations. More recently, a study conducted with football players demonstrated higher bilateral parietal alpha ERD compared to non-players when watching football actions, which was associated with visuospatial information processing^36^. In the same vein, Muraskin and colleagues (2015) compared experts and novices in baseball with a baseball-based Go/NoGo task and found that similar cortical mechanisms mediate visual prediction and expertise, which they interpreted as reflecting enhanced perception-action coupling in expert hitters^31^. Overall, these studies provided evidence of neurophysiological differences between experts and novices when processing contextual information or visual sensory information during anticipation. Yet, none of these studies have made direct comparison of the neurophysiological processes engaged when anticipating with these different information sources, nor have researchers investigated neurophysiological activity when presenting both contextual and kinematic information together. Since these two sources of information have both been shown to be relevant for accurate anticipation^22^, and in most performance environments people will have access to both contextual and visual sensory information, we decided to combine them to ensure a more representative task.

To investigate the processes supporting anticipation in cricket, we used electroencephalography (EEG) to identify the underlying neural correlates when contextual and kinematic information are integrated. We performed event-related spectral perturbation (ERSP) analyses and looked at the event-related synchronization (ERS) and desynchronization (ERD) of the EEG frequency bands. ERSP analyses were used to average the dynamic changes within the EEG spectrum as a function of time relative to an external stimulus^37^. While ERD reflects a decrease in spectral power from baseline values, and has been associated with cortical excitatory processes, ERS reflects an increase in spectral power and has been associated with cerebral inactivity or idling state^38^.

With regards to our frequencies of interest, we focused the time-frequency analyses on alpha- (8–13 Hz) and beta-band (15–25 Hz) oscillations. While beta activity is known to be related to action observation and movement planning and preparation^11,39^, alpha activity has been related to action observation and cognitive and memory performance, sensorimotor processing, visuospatial attention and movement planning^40–43^. The need to improve understanding of the neural mechanisms that support attentional processes before an action is executed, as well as the link between motor expertise and action observation, is relevant across numerous domains where fast and accurate anticipation is required.

In this experiment, expert and novice batters were required to predict the trajectory of the ball when we only presented contextual information (game situation and field setting), presented only kinematic information (video of a bowler releasing a ball), and when access to both sources of information was available. By showing these sources of information sequentially, we examine the neural signature of anticipation and how this may change as a function of access to different sources of information. We decided to choose expert batters that practiced their sport on a weekly basis, since it has been shown that visuo-motor experts who participate regularly in the sport develop more sharpened anticipatory skills compared to visual experts such as coaches or sports journalists^44^. In line with previous research, we predict that expert batters will record higher anticipation accuracy when compared to novices across all conditions. We further predict that the anticipation accuracy will be higher when both kinematic and contextual information is presented, compared to when only contextual or kinematic information is provided in both groups^22^. In regards to the EEG data, we predict that the expert group will show stronger frontal and occipital alpha ERD compared to the novice group when game situation or field setting is provided^45,46^. When the footage containing visual cues is presented, we predict that the expert group will show stronger frontocentral alpha and beta ERD compared to the novice group^11^.

## Methods

### Participants

Altogether, 12 expert cricket players (M age = 24.0 ± 5.5 years) who played at national club level and above (M competitive experience = 13.4 ± 7.6 years) and 15 novice or recreational cricket players (M age = 26.71 ± 5.8 years) with no experience of playing competitive cricket volunteered to participate. Our sample size was determined a priori based on previous literature on skilled-based differences in anticipation and on previous EEG studies with corresponding analyses^8,10,11,22,47^ reporting medium effect size, to reach a power of 0.8 to detect, with an alpha of 0.05 and an effect size f 0.25, with a mixed repeated-measures ANOVA within-between participant interaction including two groups and three measurements. A total sample size of 28 participants was required (calculated by the G x Power^48^). All the expert participants were specialist batters. A total of 5 out of 12 expert players had experience at the highest representative level nationally (minor county or above, which makes up the top three tiers of domestic cricket competition in the United Kingdom). Participants reported normal or corrected to normal vision and no history of neurological or psychiatric disease. We excluded three participants from the EEG analyses due to data loss, but they were included in the behavioral analyses. Twenty-four participants (10 expert; 14 novice) were included in the EEG analyses. We obtained informed written consent form all participants prior to the investigation. The experimental protocol and the methods were approved by the Ethics Committee of St Mary’s University, Twickenham, London, and were in accordance with the code of ethics of the World Medical Association (Declaration of Helsinki) for experiments involving human participants in research.

### Stimuli

The visual stimuli were the same as those previously employed by Runswick and colleagues^22^. These authors were the first to show expert-novice differences when using contextual information for anticipatory judgements, but they did not record EEG. Thus, we decided to use the same experimental task by modifying some parameters according to the EEG requirements, namely we used stimuli footage of the same duration, we decreased the presentation time of the stimuli, and we increased the number of trials. The video-based test stimuli were created using 10 (M age = 19.5 ± 2.5 years) county-level cricket bowlers (six fast and four spin bowlers). A camera was positioned on the batting crease at a height of 1.7 m and in line with middle stump, so that it represented the typical viewing perspective when batting. The bowlers were asked to deliver a full over (six deliveries) as they would in a game situation, yielding 60 unique deliveries. All clips were occluded immediately after ball release and had a duration of 800 ms (600 ms pre-ball release and 200 ms post-ball release). The contextual information comprised information about the field settings and game score, which included the number of overs bowled, runs scored, and wickets taken prior to seeing the delivery. In order to build congruent situations, three qualified cricket coaches viewed the non-occluded video and decided what game situation and field settings would tactically match the outcome of the delivery.

### Procedure

**Figure 1:**
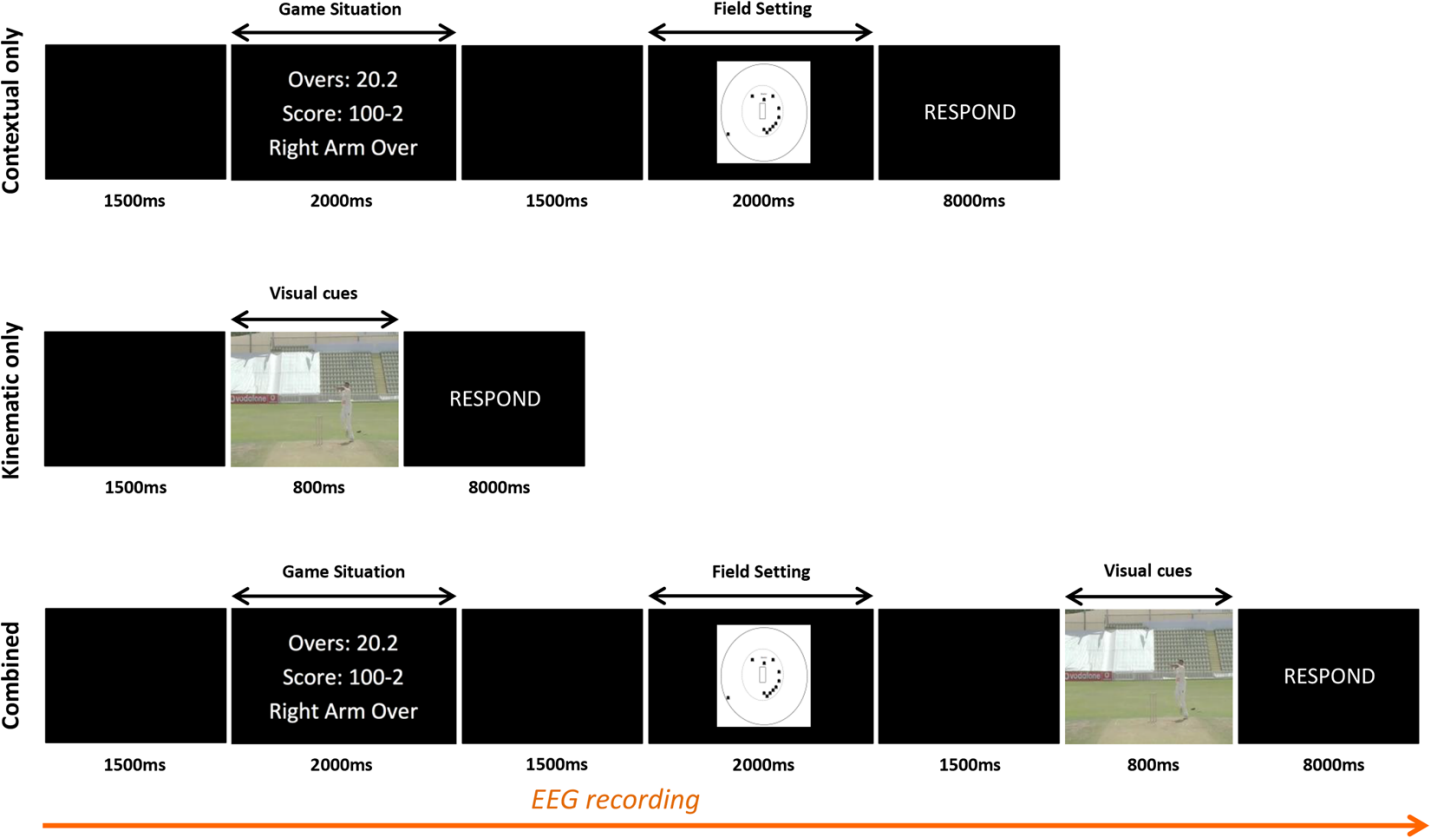
An example of a single trial for the contextual information only, kinematic information only, and combined conditions. The game situation and field setting trials are fixed images presented for 2000 ms. The visual cues footage (800 ms) shows deliveries of a bowler. Anticipation accuracy was recorded in all conditions while EEG was only collected in the combined condition.

Participants were seated in a quiet room facing a computer screen (HP ProDisplay P223a 21.5″ FHD Monitor, Palo Alto, CA) with a response sheet. Stimuli were presented on the screen using the E-Prime 2.0 software (Psychology Software Tools, Inc., Sharpsburg, PA). The experiment was divided into three conditions: context only condition; kinematic only condition; and a combined context-kinematic condition (see Fig. 1). First, participants were randomly given 24 trials of contextual (game situation + field setting) information only or 24 trials of kinematic information only (visual cues footage) to predict the ball’s future trajectory. Participants had 8 seconds to mark a cross on the response sheet after each trial displayed. After the completion of the first two conditions, we set up the EEG for the last condition, in which we presented situations where contextual (game situation + field setting) and kinematic information were combined. The images and footages were the same as those previously used in the first two conditions, but they were presented together. Since we needed more stimuli for the EEG analyses, this condition comprised of 74 trials in total. One trial consisted of an image presenting the game situation, followed by an image presenting the field setting and finally by the video footage containing kinematic information from a bowler’s delivery. In the combined condition, we instructed participants to be ready to answer after each trial, but a response was randomly asked on only 1/3 of the trials to enhance engagement. For all the situations, the contextual and the kinematic information were congruent so that the contextual information always matched the visual cues information.

### Measures and data analysis

#### Anticipation accuracy

Participants received instructions about how to use the response sheets placed in front of them before the experiment started. We provided one response sheet per trial. On the response sheet, a wicket was represented on a scaled diagram eight x smaller than game size to fit a single A4 sheet (see Runswick and colleagues^22^). We instructed participants to mark a cross on the response sheet where they predicted the ball would have passed the stumps at the batting end. The radial error corresponding to the distance between the predicted and the correct ball location was measured in centimetres and scaled back up to estimate anticipation accuracy at game scale (i.e., how far the bat would have been from the ball).

We used a two-way mixed design ANOVA to analyse the effect of Group (Expert, Novice) and Condition (Context, Kinematic, Combined) on anticipation accuracy. We controlled for normality using the Shapiro-Wilk test and for the structure of variance-covariance using the Mauchly’s sphericity test. We adjusted for multiple comparisons using the Bonferroni correction. Eta squared (η^2^) was used to measure effect size. Alpha level (*p*) for statistical significance was set at 0.05.

#### EEG recording and data pre-processing

EEG data were recorded continuously at a 2048 Hz sampling rate with a 27-channel DC amplifier (Neuro Prax EEG®, neuroConn GmbH, Ilmenau, Germany) and an impedance kept below 5 kΩ. We used BrainVision Analyzer 2.1 (Brain product, Munich, Germany) to pre-process the data. Four channels were used to send the stimuli triggers, which led to 23 electrodes montage.

After signal downsampling to 1024 Hz and filtering (0.05–40 Hz bandpass using a zero-phase shift second-order Butterworth filter, 50 Hz Notch and DC removed), we performed an independent component analysis (ICA) to remove eye blink artefacts. The C3 electrode was interpolated in all participants due to technical problems. We removed bad signal segments semi-automatically from the continuous EEG data and only epochs respecting the ±80 µV artifact rejection were accepted for further analyses.

#### EEG data analyses

EEG signals were analysed offline using Matlab (version 2018b, MathWorks, Natick, MA) and Fieldtrip toolbox^49^. The data were epoched first into 11 second long segments, starting 1.5 second prior the onset of the game situation. Spectral estimates were computed using FieldTrip toolbox using multitaper convolution method with a sliding Hanning window (4–50 Hz, 500-ms window) with a frequency resolution of 0.5 Hz and a time resolution of 10 ms. Power values were normalized relative to a baseline period (−1000 ms to −150 ms before the onset of the game situation) and finally transformed into decibel (dB) by multiplying log ratio with the factor 10^50^.

ERSP expresses the change in power (in dB) during the trial period. We first performed the ERSP analyses over the 23 electrodes to assess visually the pattern of the frequency changes.

#### EEG statistical analyses

**Figure 2:**
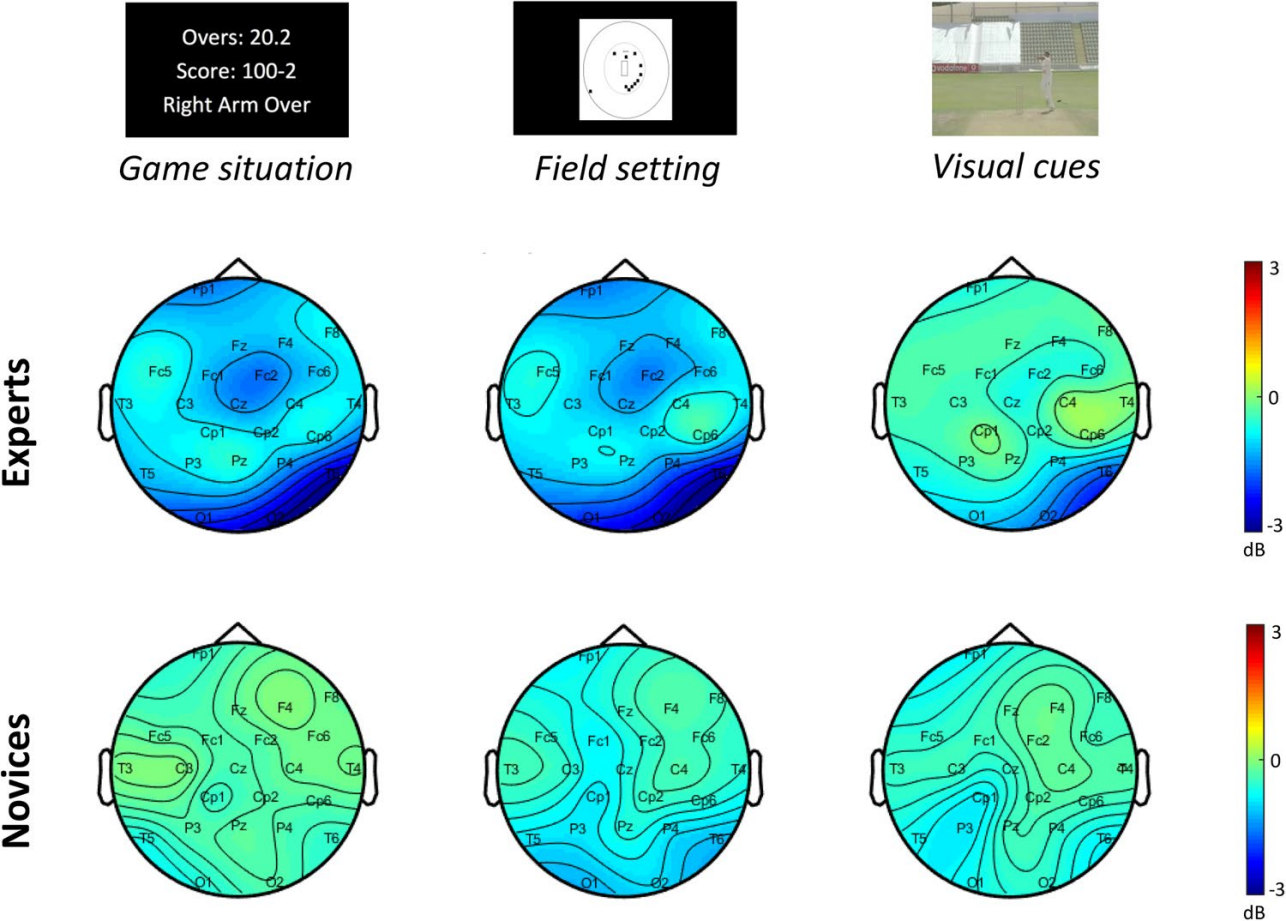
The scalp topographies averaged for each condition (i.e., game situation, field setting and visual cues) across the expert and the novice groups. Red and blue indicate positive and negative potential values in decibel, respectively.

To identify any significant differences in ERSP time/frequency data between expert and novice groups, we used non-parametric permutation tests with the Monte Carlo method^51^ for all electrodes individually. The false discovery rate (FDR) was used to control for multiple comparisons (Matlab/Fieldtrip). We decided to display the two electrodes that showed the strongest between-group difference in the alpha-band only, namely the FC2 and O2 electrodes. To allow comparison with a control electrode, we showed the ERSP of the C4 electrode. To validate the choice of electrodes, we computed the topographies averaged from 0 to 2000 ms for the game situation and the field setting images, and from 0 to 800 ms for the visual cues footages (see Fig. 2). The results of the ERSP analyses are displayed on a time-frequency plot showing points where we found significant differences at the *p* < 0.05 level. In a second analysis, we applied the same statistical procedure only for alpha (8–13 Hz) power extracted from the ERSP matrix between 0 to 2000 ms post-stimulus onset for the game situation and field setting and between 0 to 800 ms post-stimulus onset for the visual cues footage. The grey shaded areas are the periods of significant between-group differences (p < 0.05). Additionally, we calculated the mean alpha ERD over the whole epoch (2000 ms for the game situation and the field setting, 800 ms for visual cues footage). Group differences in the averaged ERSP values were calculated using an independent samples t-test (expert vs. novice).

#### Correlations between alpha ERD and anticipation accuracy

To assess the relationship between the behavioral and EEG data, we computed Pearson’s correlations between the averaged alpha ERSP values and the anticipation accuracy score of each condition (i.e. game situation, field setting, visual cues) for the FC2 and O2 electrodes.

## Results

### Anticipation accuracy

**Figure 3:**
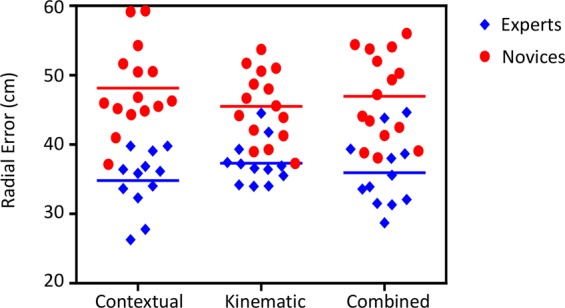
The anticipation accuracy scores for the expert and novice groups across the contextual information condition, the kinematic information condition and the combined condition. The means are represented by horizontal bars.

There was a main effect of Group (F_1,25_ = 47.02; p < 0.01; η^2^ = 0.65). The expert group was significantly more accurate at anticipating ball location compared to the novice group across all the three conditions (Mean ± SD, Expert: context 34.8 cm ± 4.3, kinematic 37.3 cm ± 3.2, combined 35.9 cm ± 5.0; Novice: context 48.1 cm ± 6.1, kinematic 45.5 cm ± 5.0, combined 47.0 cm ± 6.3). There was no main effect of Condition (F_1,25_ = 0.002; p = 0.99; η^2^ < 0.01) and no interaction between Group and Condition (F_1,25_ = 2.74; p = 0.074; η^2^ = 0.10) (Fig. 3).

### EEG

#### Group differences in game situation, field setting, and visual cues footage

**Figure 4:**
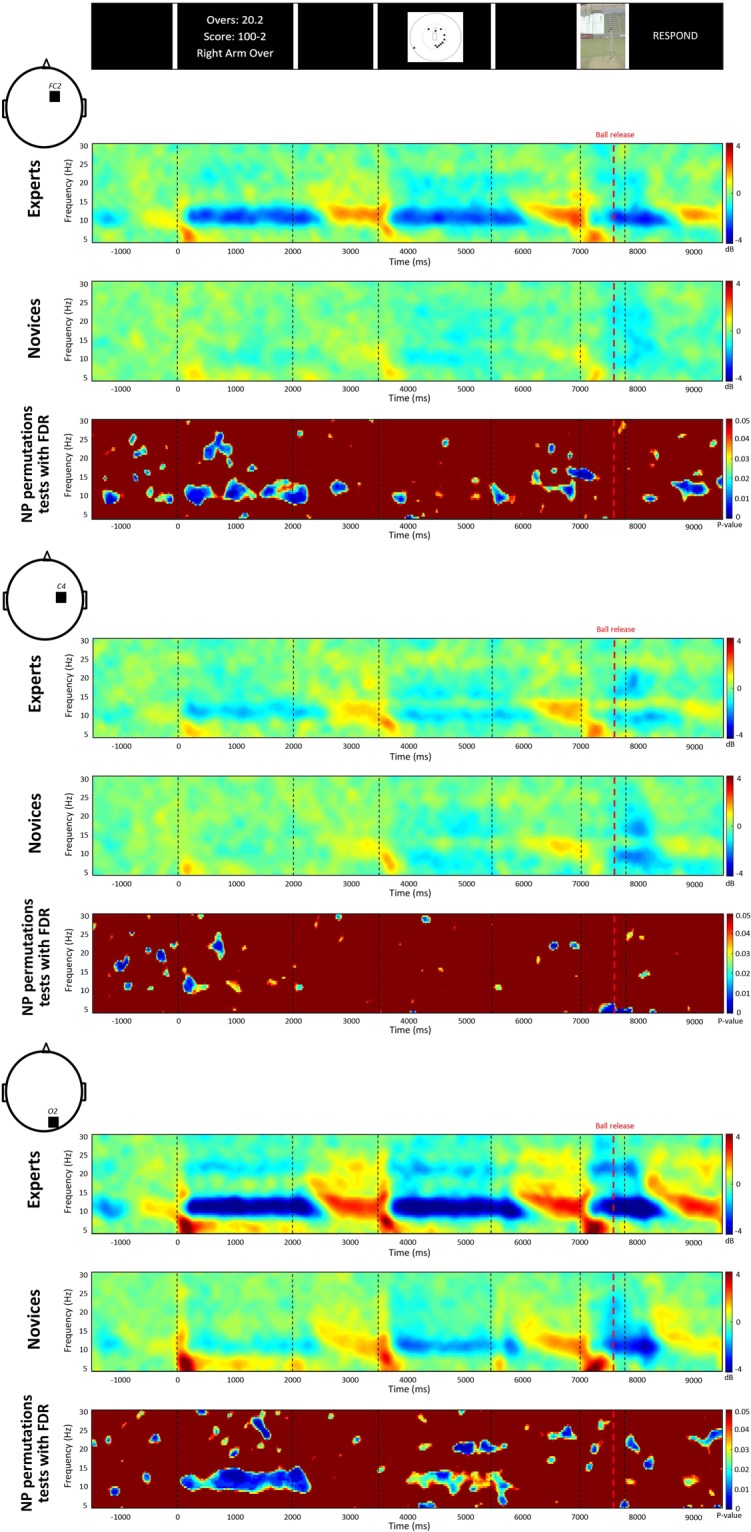
The ERSP time-frequency plots for expert and novice groups for the entire time course of the trial for the FC2, C4 and O2 electrodes. The colour blue shows a reduced power, while the colour red shows an increased power. Non-parametric permutations statistics with false discovery rate (FDR) show between-group differences on time-frequency plots (p-value < 0.05).

We report the ERSP plots of the FC2, C4 and O2 electrodes across groups in Fig. 4. In the game situation, non-parametric permutations tests showed group differences (p < 0.05) mainly in the alpha-band at the frontal (FC2) and the occipital (O2) electrode. The ERSP plots showed that the difference was driven by a stronger alpha desynchronization in the expert compared to the novice group. Similar results were reported for the field setting. In the visual cues footages, while stronger alpha ERD was found in experts than novices, this difference was not statistically significant (p > 0.05).

#### Group differences in the alpha-band oscillations

**Figure 5:**
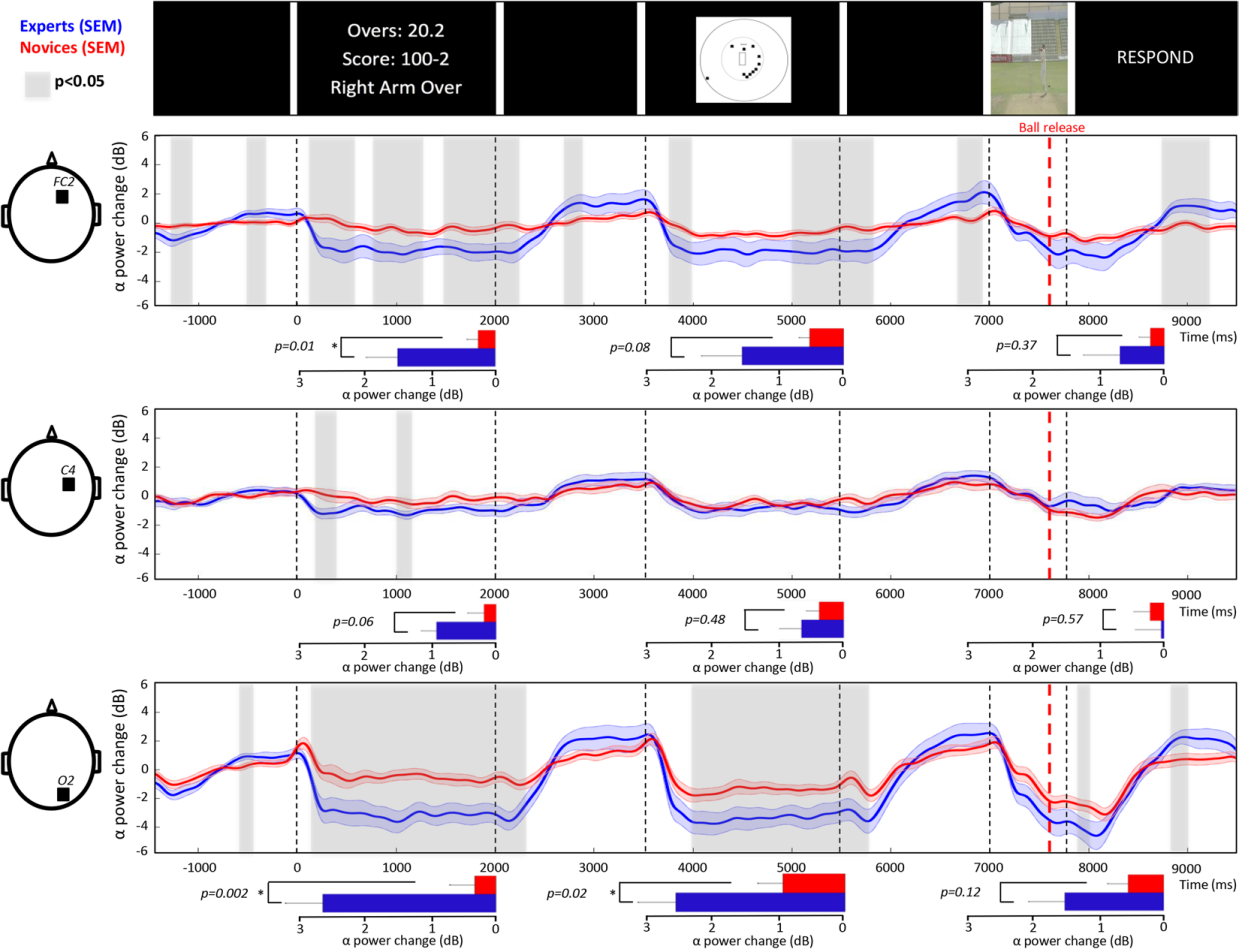
Power changes in alpha-band for the entire time course of the trial for the FC2, C4 and O2 electrodes. The expert group is represented in blue and the novice group in red with the standard error of the mean (SEM). The grey shaded areas are the periods of significant between-group differences. The bar graphs represented the alpha ERSP values averaged over the whole epoch. Error bars indicate the standard error of the mean.

The power change in decibel (dB) for the alpha-band is displayed for the entire time course of the trial for the FC2, C4 and O2 electrodes in Fig. 5. The grey shaded areas, representing the period of significant group differences, showed differences in game situation and field setting conditions at FC2, and O2 electrodes (p < 0.05).

The bar graphs depicted in Fig. 5 represent the average of the alpha power change across each whole epoch (i.e., 2000 ms for the game situation and the field setting and 800 ms for the visual cues). The unpaired t-tests showed that alpha ERD was significantly stronger in the expert group in the game situation at FC2 and O2 and in the field setting condition at O2 (mean (dB) ± SEM: FC2 game situation: expert −1.66 ± 0.53, novice −0.29 ± 0.19, t(22) = −2.73, p = 0.01; field setting: expert −1.58 ± 0.63, novice −0.53 ± 0.16, t(22) = −1.84, p = 0.08; visual cues: expert −0.67 ± 0.55, novice −0.21 ± 0.17, t(22) = −0.92, p = 0.37; C4 game situation: expert −0.94 ± 0.24, novice −0.19 ± 0.26, t(22) = −2.02, p = 0.06; field setting: expert −0.62 ± 0.32, novice −0.36 ± 0.19, t(22) = −0.73, p = 0.48; visual cues: expert −0.04 ± 0.35, novice −0.19 ± 0.23, t(22) = 0.57, p = 0.57; O2 game situation: expert −2.79 ± 0.64, novice −0.34 ± 0.36, t(22) = −3.52, p = 0.002; field setting: expert −2.72 ± 0.61, novice −1.00 ± 0.34, t(22) = −2.65, p = 0.02; visual cues: expert −1.59 ± 0.59, novice −0.57 ± 0.34, t(22) = −1.60, p = 0.12).

#### Correlations between alpha ERD and anticipation accuracy

The Pearson’s correlations showed no significant relationship between the averaged alpha ERSP values and the anticipation accuracy score on FC2 (Game situation: r = 0.29, p = 0.17; Field setting: r = 0.28, p = 0.18; Visual cues: r = 0.17, p = 0.42) nor on O2 (Game situation: r = 0.25, p = 0.23, Field setting: r = 0.28, p = 0.19, Visual cues: r = 0.16, p = 0.46).

## Discussion

We examined the neural dynamics of anticipation when expert and novice performers had to anticipate under conditions where they had access to contextual information only, kinematic information only, and a combination of these two sources. We expected to find higher anticipation accuracy in experts compared to novices in all conditions. We further predicted that anticipation accuracy would be better when we combined kinematic and context conditions compared to when only contextual or kinematic information were presented in isolation^22^. In regards to the EEG data, we expected to find expert-novice differences in alpha-band oscillation across the three situations and in beta-band oscillations in the visual cues footage^11,39,45^. Specifically, we predicted that the expert group would show stronger alpha ERD over frontal and occipital sites compared to the novice group when contextual information is presented^45,46^. In the visual cues footage, we expected the expert group to show stronger alpha and beta ERD compared to the novice group over frontocentral sites^11^.

In keeping with our hypothesis, the expert group of participants was better at anticipating across all conditions compared to the novice group. This expert-novice group difference was corroborated by the EEG analyses showing stronger alpha ERD in the expert group compared to novices over occipital and frontocentral sites, which have previously been associated with visuo-spatial attentional processes, cognitive performance, access to semantic long-term memory, as well as experienced motor representations^45,52^. Also, in line with our predictions, the expert group reported lower error scores and consequently, superior anticipation accuracy when compared with the novice group across all conditions. The information available prior to late ball flight was used more effectively by the expert group to facilitate anticipation. This finding is consistent with previous literature which has reported that skilled players are better able to use a diverse range of information sources to predict an opponent’s future actions^2,22^. For example, Runswick and colleagues^22^ showed a significant effect of expertise when participants were asked to judge the usefulness of different sources of information (namely contextual or visual kinematic cues) to anticipation. The skilled cricket players rated all of the different sources of advanced information as more useful to anticipation than the novice group.

With regards to differences between conditions, contrary to our hypothesis, we did not find a significant difference in anticipation accuracy across the three conditions. We expected accuracy to improve in the combined condition compared to the context only or kinematic only condition^22^. It is possible, however, that the participants had reached a ceiling level, whereby the usefulness of information that was conveyed when contextual and kinematic information were displayed individually provided sufficient information to enable the narrowing of probabilities and reduce error in judgments such that combining these information sources merely served to confirm the predictions that were made based on contextual or kinematic information alone.

The absence of a Group x Condition interaction is consistent with previous work in this area which has shown that anticipation accuracy is modulated in the same way in expert and novice groups. For example, Runswick and colleagues^26^ reported that while skilled players consistently outperformed their less-skilled counterparts, this difference did not differ across conditions. When the information presented was congruent with the outcome, experts simply used all information sources more effectively.

As predicted, we reported a stronger alpha ERD in the expert group compared to novices in the game situation and the field setting conditions over occipital and frontocentral sites. Alpha ERD has been associated with a release of inhibition and in turn an increased excitability of areas involved in the processing of visual stimuli^41,53^. Additionally, posterior alpha ERD is known to reflect attentional mechanisms^41,42,54,55^. In a study investigating the neural correlates underlying anticipatory mechanisms using predictive context pictures, Bidet-Caulet and colleagues^45^ reported a decrease in occipital alpha, which they associated with preparatory attentional mechanisms. It was suggested that these mechanisms, namely a pre-activation of the cortical areas that are engaged in the decisive stimulus processing, would facilitate anticipation. Our findings, which show that the expert group demonstrated stronger occipital alpha ERD than the novice group, support this hypothesis. The expert group would be more engaged in the task due to greater domain specific expertise. As mentioned previously, when discussing anticipation, the greater familiarity of the expert group with the task has recently been demonstrated with an information score showing that all information sources provided to the participants were reported as being more useful to anticipate in skilled cricket players compared to novices^22^. Nevertheless, since we did not find significant correlations between the alpha ERD and the anticipation accuracy score, we do not claim that the superior behavioral performance of our experts is entirely explained by their greater alpha ERD. Del Percio *et al*. (2019) reported the same pattern of results when they correlated the behavioral performance with the alpha ERD in football players and non-players^36^. Thus, we do not postulate the existence of a link between anticipation accuracy and alpha ERD, but we suggest that the familiarity of the experts with the information presented results in stronger attentional mechanisms which manifests itself through stronger fronto-occipital alpha ERD. However, it would have been interesting to test whether, and how, individual differences are associated with the alpha ERD. In the future, researchers may wish to collect batting averages (or other performance metrics) from participants and see how this measure of expertise correlates with the neural dynamics of the athletes.

Previous published reports have demonstrated that alpha oscillations serve as a marker for accessing long-term memory^46,56,57^. Freunberger and colleagues^46^ used real objects and meaningless objects to investigate how alpha ERD was associated with semantic access and retrieval processes in long-term memory. They observed stronger alpha suppression in the presence of real objects compared to the meaningless objects. They postulated that alpha would work as a guiding mechanism between the stimuli appearing on the screen and the knowledge stored in the semantic long-term memory. The link between alpha suppression and semantic long-term memory mechanisms could explain why our expert group displayed stronger alpha ERD while viewing contextual cues compared to novices. Due to their deep knowledge of the contextual information provided, the alpha suppression would highlight facilitated access to long-term memory, and in turn would sharpen their prediction skills. Similarly, Zion-Golumbic and colleagues^58^ presented images of famous and non-famous people in a pre-experimental session and then asked participants to assess whether the faces displayed during a subsequent experimental session were new or already watched. Their results showed larger occipital alpha ERD for famous compared to the non-famous faces in the pre-experimental session, indicating an association between alpha ERD and prior semantic knowledge. The same larger alpha ERD was found when already watched faces were presented compared to new faces, showing an association between alpha ERD and episodic memory. We suggest that the stronger alpha ERD found in the expert group would reflect a facilitated access to long-term episodic memory developed through their long-term practice of cricket. In line with our behavioral findings, as well as the information score showing that anticipatory information sources are rated as more useful by expert than novice cricket players^22^, the prior knowledge (i.e., semantic memory) and experience (i.e., episodic memory) of the expert group with the situations displayed in our task would facilitate episodic and semantic access to long-term memory when compared to the novices.

When visual information only was presented, we expected the expert group to show stronger alpha and beta ERD in frontocentral regions when compared to the novice group. While experts exhibit stronger, although not statistically significant, alpha ERD than novices over frontocentral and occipital sites, no between-group differences were found in the beta-band. Alpha and beta activity have been used as indexes for the human mirror system activity^11,47,59^, which is known to support both action execution and action observation and its activity is modulated by an individual’s motor repertoire^60,61^. Therefore, a stronger alpha ERD would reflect the access to motor representations when observing well-known and practiced actions. In keeping with our results, Wolf *et al*. (2014) did not find a significant difference in alpha ERD over fronto-parietal electrodes between expert and amateur table tennis players when watching table tennis footage, although a trend was reported for stronger alpha ERD in experts^62^. Nevertheless, others have reported significant differences in alpha ERD between experts and novices when having access to videos of well-known situations. Orgs and colleagues^47^ reported that expert dancers showed a decreased power in alpha and beta frequency bands compared to non-dancers when observing dance movement. This alpha and beta ERD was suggested to be associated with the dancers’ familiarity with the movement watched. It was argued that dancers would be able to link the movement observed with the motor representation that would match this particular movement. In the same vein, Denis and colleagues^11^ reported that experienced tennis players exhibited stronger high alpha (11–13 Hz) and beta ERD over sensorimotor areas compared to inexperienced novices when viewing a video-based anticipation task. On the basis of the findings reported in these two studies, the researchers postulated that experienced athletes would associate well-known motor representations with actions viewed in their own sport domain and that the areas involved in movement observation would be similar as those involved in movement execution. Accordingly, Quandt and Marshall (2014) demonstrated that prior experience with simple actions such as reaching, grasping and lifting actions induced alpha and beta ERD over central electrodes when observing videos clips showing these simple actions^63^. Finally, a recent study using footages of football situations demonstrated stronger bilateral parietal alpha ERD in players compared to the non-players that they associated with visuospatial information processing^36^. Nevertheless, due to the heterogeneity of the experimental designs among these studies, such as the diversity of the scenes employed, the length and number of video stimuli presented, the type of EEG analyses, the expertise level of the participants, comparing the different results is not straightforward. Finally, it is important to note that we did not include an action execution condition. We reason that the frequencies modulations would have been different if a movement had been required. Due to the constraints of our non-mobile EEG system, we were unable to design a more representative protocol with greater action fidelity.

In regards to beta ERD, it has been associated with the higher certainty of an experienced group when undertaking judgements relating to the outcome of the action. Previously, researchers have associated beta power changes with the level of certainty of an action’s outcome, postulating that stronger beta ERD would reflect greater certainty of what might happen next^64–66^. In our results, since no differences in the beta-band were reported across groups when watching the video, we suggest that the absence of beta suppression could be explained by the short duration of our video footage (800 ms) occluded just after the release, which would have resulted in higher uncertainty of the action’s outcome. Despite more accurate anticipation judgments by experts compared to novices when having access to only kinematic information, we did not capture this difference with electrophysiological measures. If we had chosen to present longer post-release timeframe, it is likely that the certainty of our expert group to predict the direction of the ball would have considerably increased, which might have led to differences in beta ERD between our two groups.

We investigated the neural processes underpinning the use of contextual and kinematic information when making anticipation judgements using a screen-based laboratory task. The task and methods we employed meant that participants did not perform a movement-based response, which could be viewed as a limitation of the study design. However, it is possible that our EEG results pattern would have been markedly different if an action had been required after or during the video footage. To control for this, and to minimise any potential confounding variables, participants were asked to remain as still as possible during the whole experiment to avoid EEG artefacts. New brain imaging technologies, such as mobile EEG^67^, are emerging and it might be that in the near future researchers would design experimental protocols in sport-specific environments to ensure greater fidelity in participant responses, such as implementing actual motor responses that mimic those employed in the field setting to test the direct association between the brain dynamics and the motor representation.

In summary, our time-frequency analyses have provided novel evidence of the neural signature underlying the anticipation of an action’s outcome based on contextual and kinematic information. We have demonstrated for the first time that experts exhibited occipital and frontocentral alpha ERD which would be linked to stronger anticipatory mechanisms and have facilitated episodic and semantic access to long-term memory when processsing contextual information during anticipation compared to novices. While cricket was used as a vehicle to study the neural processes supporting skilled anticipation, we suggest that the processes reported would be similar to those observed in numerous other professional domains such as aviation, motor racing, and law enforcement where the performer has to process different sources of information (i.e., contextual and visual) to inform anticipation judgements under strict temporal constraints.

## Data Availability

The data and codes used in this study are available upon reasonable request to the authors. The data and code used for this study comply with the requirements of the local ethics committee.
